# How we should measure orthographic depth: Or should we?

**DOI:** 10.3758/s13423-025-02831-1

**Published:** 2026-01-14

**Authors:** Xenia Schmalz, Jay G. Rueckl, Noam Siegelman

**Affiliations:** 1 Department of Child and Adolescent Psychiatry, Psychosomatics and Psychotherapy, University Hospital, LMU Munich, München, Germany; 2 Department of Psychological Sciences, University of Connecticut, Storrs, CT USA; 3 Department of Psychology & Department of Cognitive and Brain Sciences, The Hebrew University of Jerusalem, Jerusalem, Israel

**Keywords:** Orthography, Psycholinguistics, Spelling/Sound Translation, Word, Recognition

## Abstract

Cross-linguistic reading research often focuses on the effect of orthographic depth—the closeness of the relationship between print and speech. To understand its effect on reading, we need to be able to objectively quantify the level of orthographic depth of a given orthography. Previous work has suggested that different dimensions underlie orthographic depth, and it is not always clear if and how existing quantifications map onto these underlying dimensions. Here, we first examine how different measures relate conceptually to underlying theoretical dimensions. Then, we quantify the relative depth of eight European orthographies. We use existing methods and new approaches which have not been previously used to quantify orthographic depth: Distance-based measures relying on the closeness of the phonology of orthographically similar words, and mutual information, as a theory-neutral approach. The relationship between the different measures suggests that they map on two separate dimensions: the size of the orthographic units that map onto phonology and the systematicity of the mapping, in line with previous theoretical work which drew a distinction between complexity and unpredictability. The measures derived based on different theoretical assumptions largely show agreement. From a theoretical perspective, this prevents us from making differential predictions based on different approaches. From a practical perspective, this suggests that different measures may yield comparable results, as long as they tap into the same underlying dimension of orthographic depth.

Many studies on reading across languages focus on orthographic depth. Orthographic depth describes the closeness of the relationship between orthography and phonology. As an intuitive example: English is generally considered deep, because it contains many words that are not pronounced the way they are spelled, such as “Wednesday” (the letters *d* and the second *e* are silent). The English orthography has been described as an outlier (Share, 2008). As there is increasing awareness of the necessity to understand reading as a global phenomenon and to overcome the focus on reading in English (Blasi et al., 2022; Huettig & Ferreira, 2022; Share, 2021), understanding orthographic depth is an important endeavor. Depth varies substantially across orthographies (Borgwaldt et al., 2004), and has been shown to have an effect on the speed of reading acquisition (e.g., Seymour et al., 2003), the predictors of successful reading acquisition (e.g., Moll et al., 2014), and cognitive processing underlying reading in adults (e.g., Frost et al., 1987). Studies on orthographic depth have contrasted reading between pairs of closely related languages, such as German and English (e.g., Landerl et al., 1997; Schmalz et al., 2017), but also between orthographies as diverse as English and Chinese (Yang et al., 2013) and Serbo-Croatian and Hebrew (Frost et al., 1987).

Broadly speaking, the results of these studies show that reading acquisition is slower for readers of languages with deeper orthographies (e.g., Seymour et al., 2003), and that the depth of an orthography impacts the nature of the reading processes such that lexical/semantic processes or orthographic units larger than single letters (e.g., letter clusters, word bodies) play a more important role in the reading of deeper orthographies (Katz & Frost, 1992; Lallier & Carreiras, 2018; Schmalz et al., 2015; Ziegler & Goswami, 2005).

Collectively, these results suggest that orthographic depth is an important construct for both basic theory and educational practice. However, progress along these fronts may be hampered by the fact that even after decades of research, orthographic depth is still not well specified at the operational level. One reason for this situation is that the relative depth of different writing systems has often been based more on intuition than on objective quantification (but see Borgwaldt et al., 2004; Schmalz et al., 2015). Another is that although a number of ways to quantify the relationship between orthography and phonology have been proposed, these quantifications tend to be tied to particular theoretical orientations and used to quantify the orthographic–phonological properties of individual words within a language rather than the overall structure of the orthographic–phonological mapping of different orthographies. Thirdly, although orthographic depth is often treated as a unidimensional construct, there is good reason to believe it is multidimensional (Daniels & Share, 2018; Schmalz et al., 2015). To date, though, few studies have differentially examined the effects of different aspects of orthographic depth (De Simone et al., 2021; Schmalz et al., 2016, 2022; Schroeder et al., 2021), which may be partly due to our limited knowledge about how to operationalize these dimensions such that we can quantify the different sources of depth.

In this paper, we aim to advance our understanding of orthographic depth by providing an extensive overview of existing and novel operationalizations of orthographic depth, putting them into theoretical context and, when applicable, providing tentative measurements of orthographic depth according to these operationalizations. We review existing measures of orthographic depth and further introduce two types of measures which have not been previously used in the context of quantifying orthographic depth: distance-based measures, comparing the closeness of orthographic and phonological forms between word pairs, and new variants of information-theoretic measures, which capitalize on the statistics of correspondences between letters and phonemes. We further extend the existing literature by examining the different measures for eight European alphabetic orthographies from three different language groups: from the Germanic group: English, German, Dutch, Swedish, Norwegian (bokmål), and Danish; French from the Romance group; and from the Slavic group Czech.

The selection of languages was driven partly by theoretical considerations. First, by comparing English, French, and German, we aimed to reproduce previous work that suggests that these orthographies vary in terms of the underlying features of orthographic depth (Schmalz et al., 2015; van den Bosch et al., 1994). Further, it has been suggested, but not shown empirically, that Danish differs from the previously studies orthographies by having relatively simple, but unpredictable letter-to-sound correspondences (Schmalz et al., 2015). Czech has been previously studied as an example of a shallow orthography (Caravolas et al., 2005). As it mostly follows the principle of mapping single letters to single sounds (e.g., the only multiletter grapheme is *ch*; all other phonemes are represented by a single letter), it should provide a shallow baseline, as well as allowing us to verify the quantification methods in a Slavic language. The choice of orthographies was also driven by pragmatic reasons, such as the availability of corpora at the time of the project start and the authors’ language knowledge.

Given the diversity of the orthographies that have been studied under the umbrella term “orthographic depth,” it is worth considering which characteristics fall under this term, and how these can be quantified. Languages and orthographies that represent them differ on many characteristics, and some of these, like average word length, are not conceptually related to orthographic depth (e.g., Marinus et al., 2015). Many studies on orthographic depth considered alphabetic orthographies only (e.g., Schmalz et al., 2015). Others have considered the position of nonalphabetic orthographies such as Chinese (Seidenberg, 1992) along this continuum. For two reasons, we focus on alphabetic orthographies only. First, it is difficult to quantitatively compare orthographic depth in alphabetic and nonalphabetic orthographies, as the relationships between print and speech are qualitatively different. Second, nonalphabetic orthographies differ from alphabetic orthographies in terms of orthographic depth, but also in many other dimensions, such as visual complexity or the size of the inventory of characters (Chang et al., 2016; Share, 2014). While the contrast between alphabetic and nonalphabetic scripts is certainly of interest (Blasi et al., 2022; Vaid, 2022), we focus on alphabetic orthographies, where there is less variability in features other than orthographic depth. This makes it easier to compare apples to apples and is thus an important first step which we believe can inform theory and give rise to behavioral predictions.

This brings us to the next question: How do we operationalize orthographic depth? Different reading researchers may have different understandings of this concept. Historically, the Dual Route Cascaded model of single-word reading aloud (DRC) has played a large role in determining how researchers thought about the cognition of reading (Coltheart et al., 1993, 2001). Schmalz et al. (2015) proposed that different dimensions underlie what is generally referred to as orthographic depth: complexity and unpredictability. These aspects of orthographic depth were formalized through the DRC. Defining these concepts, as originally envisioned, requires an understanding of some of the assumptions that are central to the DRC. First, the model makes a distinction between a sublexical and a lexical route. The sublexical route contains grapheme–phoneme correspondences (GPCs), which are proposed to be all-or-none rules that map a letter or letter cluster (grapheme) onto a single phoneme. A defining feature is that each GPC, as implemented in the DRC, describes the spelling of a single phoneme; however, a grapheme can be multiletter (e.g., *th* → /θ/) or its pronunciation can be changed by the surrounding letters (e.g., *c* → /k/, but *c[i]* → /s/). Schmalz et al. (2015) referred to multiletter and context-sensitive GPCs collectively as *complex rules.*

As an aspect of orthographic depth, complexity is related to sublexical processing: In very shallow orthographies such as Czech or Finnish, it is almost always the case that a single letter consistently maps onto a single phoneme, regardless of the context in which it occurs. For example, in Czech, the words *reklama* /rɛklama/ [advertisement] and *karamel* /karamɛl/ [caramel] are anagrams, both orthographically and phonologically. Most letters in Czech map onto a phoneme; the only exception is the two-letter grapheme *ch*, which corresponds to the phoneme /x/. In other orthographies, multiletter and context-sensitive GPCs (in DRC-terminology) are required to predict the pronunciation of a word. For example, both in French and in English, multiletter graphemes often need to be taken into account (e.g., in French, *aient* → /ɛ/; English, *ee* → /i:/). The existence of complex rules increases the *complexity* of an orthography (Schmalz et al., 2015). In French, all words which end with the multiletter grapheme *aient* have the pronunciation /ɛ/; thus, anyone who knows this grapheme-to-phoneme correspondence can read aloud a word containing it correctly, even without ever having seen or heard this word before. A correct pronunciation for most French words will occur, assuming that the reader parses it correctly: an error may occur, for example, for the word *étaient* (/etε/; meaning “they were”) if the reader erroneously parses the five-letter grapheme *aient* into *ai, en,* and *t*, which map onto /ε/, /ɛ̃/, and /t/, respectively. *Complexity* therefore is related to ambiguity in parsing.

In the DRC model, the lexical route contains whole-word knowledge that allows readers to map entries in their orthographic lexicon to known phonological word forms. This happens in parallel to and independent of sublexical processing. In English, even multiletter graphemes often have multiple possible pronunciations, such as *ough* in “tough,” “though,” “through,” and “thought”: as the pronunciations of the vowels are different despite an almost identical orthographic context, whole-word knowledge is required. This requires the involvement of the lexical route for a correct pronunciation. Words which cannot be read aloud correctly are defined as *irregular* in DRC-terminology. On the level of a single word, irregularity is a binary construct (Coltheart, 2012). On the level of an orthography, Schmalz et al. (2015) quantified *unpredictability* as the percentage of words which are irregular.

Both for theoretical and pragmatic purposes, it is important to consider if and how the distinction between complexity and unpredictability applies outside the DRC framework. From the DRC’s perspective, taking into account whole-word knowledge is qualitatively different from taking into account information from larger sublexical letter clusters (Schmalz et al., 2022). In the DRC, lexical activation is necessary to obtain a correct pronunciation for irregular words; for complex words, sublexical processing is sufficient. Increased top-down lexical activation may be required for complex words, as compared to simple words, as well, in order to disambiguate between different parsing options (Schmalz et al., 2016); however, assuming that a reader knows the correct parsing, they can generate a correct pronunciation even while relying on sublexical knowledge only.

Evidence suggests that all-or-none GPCs may be too simplified as a model of participants’ sublexical knowledge, as there are differences, across and within readers, in their pronunciation of the same orthographic clusters (De Simone et al., 2021; Schmalz et al., 2014, 2020; Ulicheva et al., 2021). From a cognitive perspective, heterogeneity in pseudoword pronunciations can depend on reliance on sublexical units that involve more than one phoneme, such as body-rime correspondences (Treiman et al., 2003). From a linguistic perspective, this is because such sublexical units help readers to maximize the predictability of a word (Siegelman, Kearns, et al., 2020a; Treiman et al., 1995). Without relying on the concept of GPCs to understand complexity, we can understand it as the pressure to rely on larger sublexical units to reduce the uncertainty in pronunciation (Ziegler & Goswami, 2005). For example, connectionist models rely on larger units when smaller units are inconsistent, as they learn that the pronunciation of a given letter may vary depending on its surrounding letters (Perry et al., 2007; Plaut et al., 1996). In this view, the concept of a grapheme, as defined in the DRC, becomes irrelevant, because learning context-specific pronunciations may involve learning the differential pronunciation of a letter when it is part of a multiletter grapheme (e.g., *s* in *sh*) or when it is modified by surrounding letters (e.g., *g* as /dʒ/ before *i*). This questions the qualitative difference between complexity and unpredictability: irregular words, such as *book,* may become predictable once larger units are taken into account (e.g., *oo* → /u:/, which would give an incorrect pronunciation of the word “book”; -*ook* → /ʊk/).

Furthermore, in frameworks outside the DRC, the division between lexical and sublexical processing is not binary (Hendrix et al., 2019; Plaut et al., 1996). In alternative models, lexical knowledge is distributed rather than corresponding to a single node in the mental lexicon. As such, activation of parts of words leads to semantic activation (Heitmeier et al., 2023; Hendrix & Sun, 2021). The distinction between complexity and unpredictability is thus qualitative in the DRC, but quantitative in alternative models. Unpredictability can then be understood as the statistical inconsistency (even once larger units are taken into account): if the unit has more than one possible pronunciation, the reader is less likely to be able to predict the correct pronunciation.

In the following section, we describe and compute the different measures of orthographic depth. We focus on monosyllabic words only: extending the measures to polysyllabic words would both go beyond the scope of the current paper and limit the comparability between measures that can only be applied to monosyllabic words. The choice of languages was driven by pragmatic considerations, as we needed corpora that contain both the orthographic word forms and their phonological transcriptions. We used CELEX for English, German, and Dutch (Baayen et al., 1996), a resource for Slavic languages for Czech (Garabík et al., 2007), the NST Pronunciation Lexicon for the Scandinavian languages (Holmboe, 2002), and Lexique 2 database for French (New et al., 2004). Please note that the corpora were created for different purposes—we expand on this limitation in the “General Discussion” section. The preprocessing of the data are described in Supplementary Materials 1 on the project’s OSF page, where we have also uploaded a spreadsheet with the processed data that went into the current analyses. The number of monosyllabic words varied, likely both as a function of the corpus sizes and the percentage of monosyllabic words that exist in each language, from 1,155 words (Norwegian) to 8,027 words (English). For reference, the percentage of words in the corpora that were monosyllabic varied from 0.2% (Swedish) to 12% (English), though these values should not be taken at face value, as they are likely to change systematically with the overall size of the corpus. All of the orthographies examined here are alphabetic and written in the Latin script, and their corresponding languages are inflectional and Indo-European.

## What approaches exist, and what are their pros and cons?

### Qualitative descriptions

Before quantification schemes emerged for orthographic depth, the classification of depth occurred by a qualitative examination of an orthography’s characteristics. For example, a multicountry consortium ranked 13 orthographies on their depth by consensus between the participating researchers (Seymour et al., 2003). The subjective approach of an in-depth investigation of each orthography has some advantages. In particular, it can identify features of an orthography that may form different dimensions of orthographic depth. For example, an investigation and implementation of computational models in Russian showed that a prominent feature is the large degree of phonotactic dependencies, such as consonant palatalization when followed by certain vowel letters, or final consonant devoicing (Ulicheva et al., 2016). While this feature is related to phonology, it is also reflected in the orthography, where the morphological structure is preserved at the cost of transparency (cf. Serbian, where phonology is represented at the cost of morphological transparency, leading to different spellings of the almost-cognate for the word “horseshoe,” “пoдкoвa” (Russian) / “пoткoвицa” (Serbian): the third letter changes from the voiced “d” in Russian, reflecting the morpheme “pod-,” meaning “under,” to the unvoiced “t” in Serbian, reflecting its actual pronunciation, as it is followed by an unvoiced consonant, /k/). According to some conceptualizations of orthographic depth, this would make Russian a deep orthography, as some consonant letters have up to three possible pronunciations (e.g., the letter д (*d*) can be pronounced as /d/, /t/, or /d^j^/). Using an automated procedure, such as an onset entropy calculation (Borgwaldt et al., 2004, 2005; described in a later section), this type of inconsistency would inflate the depth estimate for Russian. However, on an intuitive level, this kind of inconsistency is very different from the differences in pronunciation of the cluster *ough* in the English words *tough, though, through,* and *thorough*, because in Russian, it is perfectly predictable, not only on the level of orthography-to-phonology (once we take context-sensitivity into account), but already on the level of phonology. From a theoretical perspective, a distinction between context-sensitive rules such as *c[i]* ➔ /s/ and phonotactic rules is that the latter exist in the spoken language, as children learn phonotactic constraints well before they start learning to read. It is still largely an open question if and how the cognitive reading system is affected by orthographic irregularities that reflect phonotactic constraints. Thus, a qualitative investigation should allow researchers to identify features that may or may not be associated with depth and provide a framework for which features should be captured in an automated quantification measure.

### Basic approaches

Subsequent cross-linguistic studies have attempted to use more objective measures. Objective quantification methods (such as those we discuss below) have the advantage of being easier to compute, as researchers with no knowledge of a language can obtain an estimate of its orthography’s depth, as long as they have access to the necessary language-level information from an appropriate corpus, containing the orthographic and phonological word forms. Furthermore, measures that are objective and computable are more reproducible, as researchers following the same computational methods should obtain identical or similar results.

Already without relying on any particular theoretical framework, we can perform simple and automatable calculations on language statistics which intuitively reflect something about orthographic depth. As a shallow orthography is often defined as an orthography where a letter consistently corresponds to a phoneme, we can calculate an easy proxy measure: the percentage of words which have the same number of letters as phonemes (e.g., *cat*, /kæt/, which has three letters and three phonemes, as opposed to *that*, /θæt/, which has four letters and three phonemes). In a perfectly shallow orthography, the percentage of words with the same number of phonemes and graphemes should be 100%. Note that finding a high percentage of words with the same number of phonemes and graphemes is not sufficient to show that an orthography is shallow, as a single-letter grapheme may have different pronunciations across words (e.g., *mint*, /mɪnt/ and *pint*, /pɑɪnt/, in English).

For the next measure, we count the number of unique grapheme–phoneme pairings. For example, when it is not part of a multiletter rule, *t* corresponds to /t/, while *c* may correspond either to /k/ (as in *cat*) or to /s/ (as in *ceiling*). Thus, there is only one letter-phoneme pairing for the grapheme *t*, but two pairings for *c*. In a perfectly shallow orthography, the total number of letter-phoneme pairings should equal the number of letters. A complicating factor for this approach is the presence of multiletter graphemes. At least for monosyllabic words, grapheme parsing can be, in principle, automated (Coltheart et al., 1993; Pritchard et al., 2016); however, ambiguities remain, for example, when it comes to silent letters. Here, we rely on a semiautomated procedure (see Supplementary Materials 2 on the project’s OSF page, https://osf.io/fmxy8/. for more details). Following such coding, we can extract both the total number of graphemes, and the number of grapheme–phoneme pairings. Table 1 presents these measures for the monosyllabic words from our corpora. Table 1 also presents the total number of letters (including letters with diacritics; e.g., *ř*, *á* for Czech) and phonemes in each orthography. These are not measures of orthographic depth but are presented for illustrative purposes. The total number of graphemes (which includes multiletter graphemes, such as *ch* in Czech) is also provided. In all cases, the total number of graphemes is larger than the number of letters. The ratio of graphemes to letters corresponds to our intuitive understanding of orthographic depth, with the smallest ratio for Czech (45:42) and the largest ratio for English (147:26).

**Table 1:** Basic orthographic descriptives that may act as proxies for depth

Language	Total number of letters	Total number of graphemes	Total number of phonemes	Percentage of words with same number of letters and phonemes	Number of unique grapheme- phoneme mappings
Czech	42	45	42	92	71
Danish	29	75	46	79	129
Dutch	26	84	44	43	151
English	26	147	46	22	262
French	33	117	39	6	185
German	30	97	47	29	150
Norwegian	29	71	49	57	109
Swedish	29	89	41	64	142

The last two measures in Table 1 are the potential proxies of orthographic depth, as described above. The percentage of words with the same number of letters as phonemes presumably links to the concept of complexity, as multiletter rules (or, more rarely, instances when a single letter maps onto two phonemes, such as *x* → /ks/) affects this measure. Indeed, languages with orthographies that are considered complex (French, English) have the lowest percentage of words with the same number of letters and phonemes. The number of unique grapheme–phoneme mappings potentially conflates unpredictability and complexity, because a letter’s pronunciation may either change in an unpredictable manner (e.g., for etymological reasons, e.g., *g* in *gift* vs. *gist*), or due to a context-sensitive rule (e.g., *c* in *circus* vs. *cortex*).

### Rule-based approach: Complexity and unpredictability

In the introduction section, we describe the concepts of complexity and unpredictability, which have been originally derived from a computational implementation of the Dual Route theory (Coltheart et al., 2001). To quantify related measures for our orthographies, we relied on existing implementations for English, French, German, and Dutch, and implemented novel versions for Czech and the Scandinavian orthographies. The language-specific files can be downloaded from the project’s OSF page. The number of complex (multiletter and context-sensitive) rules serves as an indicator of complexity, and the percentage of irregular words quantifies unpredictability.

In Table 2, we present the number of the different types of rules and the percentage of irregular words for Dutch, English, German, and French from Schmalz et al. (2015), as well as from a novel implementation of the DRC in Czech, Danish, Norwegian, and Swedish. This table shows Czech as a very shallow orthography (only 1% of words are irregular, mostly loanwords such as “job”, which is pronounced /dʒop/ rather than /jop/). Czech has few multiletter and context-sensitive rules; the relatively high number of single-letter rules, as well as most of the context-sensitive rules, result from phonotactic constraints, such as the devoicing of final consonants or consonant assimilation (for a discussion of phonotactic rules in Russian, another Slavic language, see Ulicheva et al., 2016). Conversely, in Danish, over half of the words are irregular: this is mostly due to unpredictabilities for silent letters (e.g., “gold” [barren] pronounced /gʌl/ versus “bold” pronounced as /bʌld/ [ball]).

**Table 2:** Complexity and unpredictability according to the DRCs

Language	Number (%) of single-letter rules	Number (%) of multiletter rules	Number (%) of context-sensitive rules	Percentage of irregular words
Czech	66 (75%)	2 (2%)	20 (23%)	1.0
Danish	36 (35%)	37 (36%)	29 (28%)	56.9
Dutch	51 (49%)	42 (40%)	11 (11%)	6.3
English	38 (8%)	161 (34%)	272 (58%)	16.9
French	46 (14%)	218 (64%)	76 (22%)	5.6
German	44 (34%)	55 (42%)	31 (24%)	10.5
Norwegian	27 (33%)	40 (49%)	15 (18%)	23.1
Swedish	28 (28%)	39 (39%)	34 (34%)	19.1

The results in Table 2 extend those previously reported by Schmalz et al. (2015) to a greater number of orthographies. The addition of the Scandinavian languages and Czech shows that Czech is shallow, both in terms of complexity and irregularity. The Scandinavian languages, conversely, have a relatively high proportion of irregular words. This is particularly striking in the case of Danish. The number of complex rules in Danish is relatively low, corresponding approximately to those of the other Germanic orthographies (excluding English). Thus, Danish is likely to be high in unpredictability but low in complexity. The French orthography, which is high in complexity, but low in unpredictability, is another case where the two dimensions dissociate.

Despite these insights, this approach to quantifying complexity and unpredictability comes with several potential caveats. First, the measures are derived from the DRC. On the one hand, this is an advantage, as it ensures a close link between a theoretical framework and the quantification measures. On the other hand, the notion that the sublexical route consists of rules is controversial (Perry et al., 2010; Plaut et al., 1996). Questioning the psychological reality of GPC rules calls into question the validity of these measures. More broadly, a quantification scheme based on a single model is not sufficient to fully understand the concept of orthographic depth. For this area of research to progress, it is essential to consider definitions and quantifications that are derived from alternative theoretical frameworks. These might show substantial overlap with the DRC-derived measures, in terms of showing overlapping dimensions and identical rankings of orthographies on each of these dimensions. Alternatively, it is possible that some dissociations will be found. This would lead to further research questions, which would inform theories of reading aloud as well as theories of cross-linguistic differences in reading.

Second, there is no unambiguous way to determine the GPC rules. Even in the implemented versions of the DRC in English (Coltheart et al., 2001), German (Ziegler et al., 2000), French (Ziegler et al., 2003), Italian (Mulatti & Job, 2003), and Dutch (Schmalz et al., 2015), the GPC rules have been hand-picked by the modelers (but see Coltheart et al., 1993; Pritchard et al., 2016). This involves arbitrary decisions. In English, for example, the modeler needs to decide whether to include the rule *g[i]* → /dʒ/ (as in *gist*), or to determine the pronunciation of the grapheme *g* in words containing the cluster *gi* by the single-letter rule, *g* → /g/ (as in *gift*). Including the context-sensitive rule would allow the model to read aloud the word “gist” correctly, but would yield the word “gift” irregular, while excluding it would yield the word “gift” regular and the word “gist” irregular. If the choice is left to the modeler’s discretion, a tendency to include context-sensitive rules will systematically decrease the unpredictability of an orthography, while increasing its complexity. With a sufficient number of context-sensitive rules, up to 100% of all words can be made regular: for example, the word “yacht” would become regular if we include the context-sensitive, muti-letter rule *[y]ach* → /ɔ/. In this extreme example, most researchers would intuitively reject the possibility that such a rule has psychological reality; however, in the case of the *g[i]* context-sensitive rule, this is not clear. In the absence of a set of objective criteria for defining grapheme-correspondence rules, therefore, it is not clear whether the complexity and unpredictability measures derived from DRCs (and other similar approaches) across languages are truly comparable to each other.

### Statistical measures

Not all theories hold that phonological decoding is accomplished via rules. Although non-rule-based theories vary in mechanism (cf. Glushko, 1979; Kay & Marcel, 1981; Plaut et al., 1996; Seidenberg & McClelland, 1989), they are generally agreed on two points that are particularly relevant to the notion of orthographic depth. First, while rule-based theories usually (although not necessarily) assume that the statistical structure of the orthographic–phonological (O-P) mapping that drives decoding are on the level of grapheme–phoneme co-occurrences, these alternative theories tend to focus on co-occurrences at larger grain sizes (see below). Second, in the rule-based framework, if a given grapheme has two or more possible pronunciations, only the most frequent one will be stored in the cognitive system as a GPC rule and information about less frequent pronunciations will be discarded. In contrast, non-rule-based frameworks hold that information about these less frequent correspondences is maintained and that the probability that an orthographic unit will be pronounced in different ways is graded by the relative frequency of their correspondences with that orthographic unit. Given the differences between these frameworks, the measures of orthographic depth derived from the DRC framework may not capture the structure of the O-P mapping hypothesized to be important by the alternative approach. However, as discussed below, the graded strength of print-to-speech correspondences and the relative size of units in an orthography can be quantified via other statistical measures (Borgwaldt et al., 2004; Siegelman, Kearns, et al., 2020a, Ziegler et al., 1997).

### Consistency

One quantification of the graded nature of O-P knowledge that has played a prominent role is described in the literature on word and nonword reading and is referred to as *body consistency*. The body of a monosyllabic orthographic word form includes the vowel and any subsequent consonant(s). When it was first introduced (Glushko, 1979), body consistency was treated as a binary construct: a consistent body is pronounced the same way in all the words that contain it (e.g., -*ill* in *mill*, *hill*, *fill*), whereas an inconsistent body is pronounced differently across words (e.g., -*int* in *mint* and *pint*)*.* However, it is now common to treat consistency as a graded measure, such that the consistency of a word is given by the ratio of its *friends* (words that share an orthographic body and rhyme) to *enemies* (words that share an orthographic body and do not rhyme). While body consistency is typically calculated at the level of a single word within a language, it can also be averaged across all monosyllabic words in a given orthography. The averaged value may serve as a statistical quantification of orthographic depth: low average consistency indicates high ambiguity in assigning a pronunciation to an orthographic unit, characteristic of a deep orthography (Peereman & Content, 1999).

Previous studies have mostly focused on consistency on the level of the body (e.g., Jared, 2002; Ziegler et al., 1997). However, consistency can be calculated for other orthographic units, including in particular at the level of a grapheme (*th* in thyme vs. thing) or syllable (*na* in nation vs. national). The focus on bodies is likely a reflection of the development of this measure in the context of the English orthography. In non-European orthographies, the natural phonological division of monosyllabic words follows different patterns (e.g., Kim, 2008; Share & Blum, 2005); as the body is prominent in monosyllabic words, its role may further be diminished in languages with a higher percentage of polysyllabic words.

Here, in addition to the classic measure of body consistency, we also computed consistency at the grapheme level (focusing specifically on vowels, which are generally the most inconsistent graphemes, at least in English). This enables a comparison of the extent to which O-P unpredictability is different at the smaller grain size of the individual vowel grapheme, versus at the larger grain size that includes the vowel and its consonantal context (Treiman et al., 1995). Table 3 presents both the body consistency and vowel consistency measures for our orthographies.

**Table 3:** Body and vowel consistency

Language	Vowel consistency	Body consistency
Czech	0.98	0.99
Danish	0.49	0.83
Dutch	0.89	0.97
English	0.64	0.89
French	0.87	0.93
German	0.72	0.93
Norwegian	0.49	0.88
Swedish	0.54	0.92

As shown in Table 3, the Czech orthography has the highest consistency, both of the vowel pronunciation and of the body pronunciation. Interestingly, the Germanic languages are generally low in terms of vowel consistency. This is likely to be, at least in part, due to inconsistency in vowel lengths, as Germanic languages have both long and short variants of the same vowel (e.g., in German, *Mond* (moon) and *blond* (blonde) have the same body, but the /o/ is pronounced as long in the first example and short in the second). Danish has the lowest values of consistency, even compared to English. Comparing this to the results of Table 2, this suggests that the consistency measures above may have a stronger relationship to unpredictability rather than complexity. This is expected given how consistency is computed—vowel consistency, in particular, is computed given the probability of a *grapheme* mapping into a phoneme (that is, after grapheme segmentation). It is also noteworthy that the differences between the languages are much more pronounced with regard to vowel consistency than body consistency. Overall, body consistency is lower than vowel consistency: thus, the uncertainty associated with individual vowel graphemes is diminished when the context that those graphemes appear in is taken into account. By calculating body consistency instead of vowel consistency, we are taking into account more information. Considering larger units is thus likely to increase consistency across in any orthography (provided it is not perfectly shallow to begin with).

### Entropy

Using vowel consistency as a starting point, we can use further calculations to refine statistical measures of orthographic depth. Specifically, in information theory, uncertainty is indexed by entropy: the more possible outcomes, and the more uniform the probability distribution of those outcomes, the more uncertain the outcome and the greater the entropy. In the context of quantifying O-P systematicity, entropy can be applied to overcome one drawback of the consistency measure: the same consistency value can reflect different scenarios (Protopapas & Vlahou, 2009). If we take the example of body-consistency, a body *X* with a body-rime consistency value of 0.25 could have one word where the body has Pronunciation *P*_1_, and three words which have a Pronunciation *P*_2_. Alternatively, there could be four words with body *X*, each with a different pronunciation (*P*_1_ to *P*_4_).

Mathematically, entropy *H(X)* is calculated as:

1 $$H(X) = -{\sum }_{i=1}^{n}(p({x}_{i}) {log}_{2}(p({x}_{i}))$$

To illustrate, in the alternative scenarios described above where the body *X* has a consistency of 0.25, we can use entropy to quantify uncertainty across the *n* possible pronunciations by calculating the probability of each possible pronunciation *P*_*i*_ and summing the products of these probabilities with their logarithms. In the case where the body *X* is pronounced as *P*_1_ in one word and *P*_2_ in three other words, the entropy of X would be:

$$H\left(X\right)= -\left(0.25\times {\mathrm{log}}_{2}\left(0.25\right)+0.75\times {\mathrm{log}}_{2}\left(0.75\right)\right)=0.81$$

In contrast, in the case where the body *X* is pronounced differently in each of four words (*P*_1_ to *P*_4_), the entropy of* X* would be:

$$H\left(X\right)= -\left(0.25\times {\mathrm{log}}_{2}\left(0.25\right)+0.25\times {\mathrm{log}}_{2}\left(0.25\right)+0.25\times {\mathrm{log}}_{2}\left(0.25\right)+0.25\times {\mathrm{log}}_{2}\left(0.25\right)\right)=2$$

Thus, larger values are associated with higher uncertainty. As with consistency, entropy can be calculated for different units (e.g., grapheme, body) and for individual words. Averaging the entropy value for all words in an orthography gives an indication of the orthography’s depth.

Measures of entropy have been used in multiple ways to capture O-P systematicity and their behavioral impact—for example, to quantify differences in O-P systematicity across items within a language (Siegelman, Kearns et al., 2020a, Siegelman, Rueckl et al., 2020b) and to compare behavioral reading-aloud data across reading acquisition and across languages (De Simone et al., 2021; Schmalz et al., 2020). Most relevant to the focus of this paper, Borgwaldt et al. (2004) used *onset entropy* as a measure of orthographic depth to compare five alphabetic writing systems. For each writing system, they first computed relevant co-occurrence statistics—the number of words in a corpus that started with a given letter and a given phoneme. These frequency tallies were then transformed to probabilities (i.e., the probability of a given phoneme given a particular initial letter) and the entropy of the probability distribution for the pronunciation of each initial letter was computed using Equation 1 above. To derive a single value characterizing the depth of each writing system, the entropies of the individual letters were summed, with the entropy for each letter weighted by the probability that a word begins with that letter. We calculated Borgwaldt’s onset entropy (see Table 4) for our orthographies, based on the corpora of monosyllabic words used for all other analyses reported here. The results are displayed in Table 4. As might be expected, Czech has the lowest onset entropy and English the highest.

**Table 4:** Onset and vowel O-P entropy for monosyllabic words

Language	Onset entropy	Context- independent	Context-dependent: Onset-conditional	Context-dependent: Coda-conditional
Czech	0.16	0.06	0.01	0.03
Danish	0.49	1.37	0.83	0.37
Dutch	0.44	0.35	0.19	0.06
English	0.57	0.94	0.63	0.22
French	0.4	0.34	0.1	0.11
German	0.54	0.66	0.45	0.15
Norwegian	0.50	1.2	0.86	0.23
Swedish	0.51	1.05	0.75	0.16

Although already potentially informative regarding differences in orthographic depth (Borgwaldt et al., 2005; Ziegler et al., 2010), this measure is limited to the first letters and phonemes of each word. This has the advantage of computational convenience and allows for the inclusion of polysyllabic words. However, word initial positions may not reflect O-P systematicity in the writing system as a whole. For example, many of the O-P ambiguities in English involve the pronunciation of vowel graphemes, which mostly occur in middle positions of words (Treiman et al., 1995).

In contrast to Borgwaldt et al.’s focus on initial positions, Siegelman and colleagues (2020) quantified vowel entropy for English monosyllabic words as an item-level measure of O-P systematicity. In addition, they extended the approach by also quantifying context-dependent vowel entropy given different orthographic environments as a way of mapping O-P systematicity at different grain sizes. When calculating context-independent vowel entropy, they took into account the percentage of times that a given grapheme co-occurs with a particular phoneme, regardless of the orthographic environment of the vowel unit (i.e., across all words). For context-dependent entropy, they calculated the percentage of times that a vowel grapheme co-occurs with a phoneme when it is either preceded (conditional on onset) or succeeded (conditional on coda) by particular consonant graphemes. In their analysis, Siegelman, Kearns et al. (2020a), Siegelman, Rueckl et al. (2020b) calculated these measures at the level of individual vowel graphemes (focusing on monosyllabic words, where the CV-structure is relatively easy to define). Analysis of data from the English Lexicon Project (Balota et al., 2007) suggested that entropy (at different grain sizes) significantly predicted RT and accuracy in visual word recognition tasks.

Here, to extend these measures as indicators of orthographic depth (akin to Borgwaldt et al.’s onset entropy), we computed the three entropy values for each vowel and each language, and then averaged each measure across all vowels (weighted by the vowel frequency). In Table 4, we report the average entropy values, both context-independent, and context-dependent (i.e., onset-conditional and coda-conditional), for our orthographies of interest. As noted above, we also report Borgwaldt’s onset entropy (not to be confused with onset-conditional vowel entropy).

The entropy values converge with the quantification methods so far, with Czech as the shallowest orthography and Danish and English as the deepest in two out of three measures. In the onset entropy measure, English comes out as being deeper than Danish, while the reverse is the case for vowel entropy. This means that inconsistencies in Danish are driven to a larger extent by vowels than onset letters, compared to English. Context-independent and coda-conditional entropies are in line with the vowel consistency and body consistency measures above. As noted above, coda- and onset-conditional entropies indicate the extent to which taking into account the consonants succeeded or preceding the vowel decrease the variability of vowel pronunciation (Treiman et al., 2003, 2006). In all languages under investigation, taking context into account decreases the entropy. Furthermore, in all but two orthographies, the coda reduces entropy to a greater extent than the onset. The two exceptions are Czech (which has a very low vowel entropy to begin with) and French (where the reduction in uncertainty is very similar across the two conditional measures).

### Efficiency through mutual information

The statistical measures so far involve an explicit decision about the grain size in which orthographic depth is computed (e.g., focusing on onsets vs. vowels; using context independent vs. conditional probabilities constrained by context). Information theory, however, offers additional tools that can be used to circumvent the need to make such decisions. Specifically, another statistical measure we consider is *efficiency*, which can be calculated by relying on another concept from information theory, *mutual information*. To our knowledge, this measure has not been applied to quantifying orthographic depth. Unlike the statistical measures introduced in the previous sections, we use mutual information to examine the relationship between print and speech sounds for the whole word, as opposed to only specific parts of the word (e.g., onset, body, or vowel). Conceptually, mutual information allows us to quantify, for an entire word, to what extent the actual pronunciation deviates from what would be expected based on the orthography.

Akin to entropy, mutual information is a construct from information theory and is thus a generic measure that has been applied in a variety of psychological and non-psychological domains (Feldman, 2021; Vicente et al., 2011). Generically, mutual information is a measure of the mutual dependence between two random variables X and Y—the extent to which the value of Y can be predicted on the basis of X. In the present context, X and Y are the orthographic and phonological domains and mutual information is a measure of the degree to which the presence of a given phoneme (or other phonological unit) is predictable from the presence of a given letter (or another orthographic unit). Put another way, mutual information is a measure of the degree to which knowing how a word is spelled reduces uncertainty about how that word is pronounced.

Mutual information can be calculated in a variety of mathematical equivalent ways (Cover & Thomas, 2006). One formulation that is particularly useful for present purposes characterizes the mutual information between two variables as the difference between the conditionalized and unconditionalized entropy of one of those variables. With regard to the mapping between orthography and phonology, we can write this as

2 $$I\left(\mathrm{O};\mathrm{P}\right)= \mathrm{H}\left(\mathrm{P}\right)-\mathrm{H}\left(\mathrm{P}|\mathrm{O}\right)$$

In this equation, H(P) is uncertainty about the identity of a phonological unit in the absence of information about the written form and H(P|O) is uncertainty about that phonological unit given knowledge of the corresponding orthographic unit. Mutual information *I(O;P)* is the difference between uncertainty in the identity of a phonological unit with or without information about the written form; equivalently, it is the *information gained* by knowing how that phonological unit is written. For a given value of phonological entropy (H(P)), mutual information is maximal when phonology is completely predictable from orthography (in which case H(P|O) = 0 and I(O;P) = H(P)) and it is 0 if the O and P distributions are unrelated (in which case H(P|O) = H(P)).

As demonstrated above, the degree to which phonology is predictable from orthography depends in part on the size of the orthographic and phonological units (e.g., graphemes vs bodies; phonemes vs rhymes). Our initial mutual-information analysis assumed that the relevant units are letters and phonemes and that these units are aligned on a one-to-one, left-to-right basis. Thus, for example, for the word “tip” the letter-phoneme correspondences are *t* → /t/, *i* → /ɪ/, and *p* → /p/. In subsequent analyses, we computed mutual information based on correspondences between bigrams and biphones and between graphemes and phonemes.

In a perfectly transparent writing system, each letter would be mapped to exactly one phoneme and each phoneme would be spelled in exactly one way. The analyses reported here quantify the deviance between various writing systems and a completely transparent system defined in this way. For each language, we first computed its phonological entropy based on the frequency distribution of phonemes in the language (i.e., H(P). Then, to compute H(P|O), we counted the number of times that each letter co-occurred in the same position as each phoneme across all the monosyllabic words in the dictionary for that language. Multiple tokens of the same letter (e.g., the d’s in *dad*) were treated as separate observations, and for words composed of more letters than phonemes the final unmatched letters (e.g., *e* in *bite*) were discarded.^1^

Mutual information (I(O;P) was computed by subtracting H(P|O) from H(P). Note that the resulting measure is dependent not only on H(P|O), but also on H(P): it tells us about the extent to which uncertainty in phonology is reduced once we take into account orthography. Because discussions of orthographic depth have generally not been concerned with baseline differences in the distribution of phonemes, to compare writing systems we calculated the proportion of entropy accounted for, or the *efficiency* with which orthography predicts phonology, by dividing the information gained (I(O;P)) by the phonological uncertainty absent any orthographic information (H(P)). The results are presented in Table 5 (top section, “Letters/Phonemes”) and are broadly similar to those of the analyses reported above, with Czech having the highest efficiency and English and German the lowest.

**Table 5:** Mutual information and related values

Units	Language	H(P)	H(P|O)	I(O;P)	Efficiency
Letters/Phonemes	Czech	4.85	0.48	4.37	0.90
	Danish	4.75	1.11	3.64	0.77
	Dutch	4.61	1.72	2.89	0.63
	English	4.88	2.17	2.71	0.56
	French	4.72	1.64	3.08	0.65
	German	4.64	2.04	2.59	0.56
	Norwegian	4.81	1.02	3.79	0.79
	Swedish	4.75	1.05	3.70	0.78
Bigrams/Biphones	Czech	4.85	0.12	4.74	0.98
	Danish	4.85	0.46	4.39	0.91
	Dutch	4.61	0.54	4.07	0.88
	English	4.88	0.94	3.94	0.81
	French	4.72	0.57	4.14	0.88
	German	4.64	1.05	3.59	0.77
	Norwegian	4.81	0.42	4.40	0.91
	Swedish	4.75	0.45	4.30	0.91
Graphemes/Phonemes	Czech	4.85	0.29	4.56	0.94
	Danish	4.80	0.82	3.98	0.83
	Dutch	4.63	0.24	4.39	0.95
	English	4.92	0.57	4.35	0.88
	French	4.25	0.59	3.67	0.86
	German	4.66	0.39	4.26	0.92
	Norwegian	4.85	0.46	4.38	0.90
	Swedish	4.76	0.40	4.36	0.92

With regard to orthographic depth, efficiency and mutual information are more closely related to unpredictability than to complexity. It is important to note, however, that nonetheless complexity has an impact on these measures. One reason is that, as discussed above, languages differ in the extent to which orthographic–phonological correspondences involve orthographic units larger than individual letters (e.g., letter clusters; word bodies). Another is that, given the one-to-one, left-to-right process used to align orthographic and phonological units, the presence of multiletter units (as well as other features such as silent letters) can result in misalignments that lower the apparent efficiency of the writing system. For example, if we take the word “chat”, with letters and phonemes as the relevant units, the O-P correspondence for the third position is *a* → /t/.

To explore this, we conducted two additional analyses, one assuming that the relevant units are bigrams and biphones, the other assuming that the units are graphemes and phonemes. The results for the bigram/biphone analysis are reported in the middle section of Table 5. We can see an overall decrease in H(P|O) and an increase in mutual entropy/efficiency associated with increasing the unit size in this way: Unsurprisingly, across languages, examining O-P relations at a larger grain size (bigrams rather than unigrams) reduces inconsistency (i.e., increases efficiency). The third analysis was based on the grapheme parsing used for the analyses reported in Tables 1 and 2. Overall, the grapheme parsing reduces H(P|O) and thus increases mutual information and efficiency, even compared to the bigram calculations. Broadly speaking, the ranking of the orthographies follows a familiar pattern to the ones observed in earlier sections, with Czech showing the smallest values of H(P|O) and largest values of I(O;P) and efficiency, and English showing high values of H(P|O) and small values of I(O;P) and efficiency.

### Distance-based measures

Many of the measures reviewed above are contingent on decisions to be made by the researcher. First, to compute rule-based and most statistical measures, one needs to specify the right segmentation of a word to GPCs. The process of GPC segmentation is not only labor intensive, but also often requires non-trivial decisions. Such decisions should, ideally, reflect the psychological reality of the units which underlie reading processes—however, we still know relatively little about psychologically real units. For example, details such as the processing of split grapheme (e.g., *a_e* in “cake”) are implemented in different ways across models, and there is no consensus about whether they constitute a single unit or an inconsistent single-letter correspondence and a silent *e*. An in-depth discussion of such choices in the case of English is presented in Kearns (2020).

Second, as noted above, statistical measures can be computed for different units and on multiple grain sizes (e.g., on letters versus graphemes; for the vowel only versus the body level, using unconditional versus conditional statistics), meaning that there are multiple measures associated with each word representing uncertainty over different units. This issue is further exacerbated in multisyllabic words (Chateau & Jared, 2003; Chee et al., 2020). This poses a challenge for summarizing a single statistic per writing system.

The new measure presented in the current section was designed with the goal to circumvent these issues. This measure is inspired by a series of recent studies aiming to quantify a word’s orthographic-*semantic* consistency (OSC; Marelli & Amenta, 2018; Marelli et al., 2015; Siegelman et al., 2022). In these studies, a measure of OSC is extracted by examining the mean *semantic similarity* of a word to its *orthographic neighbors*. The idea behind this quantification is that a word that is more orthographically-semantically consistent should be more similar semantically to words to which it is similar orthographically (i.e., its orthographic neighbors). And whereas the exact operationalization varies across studies (Siegelman et al., 2022), they all share a basic premise: They all quantify a word’s consistency in a quasi-regular mapping by examining the similarity in one dimension (e.g., semantic similarity) between a word and its neighbors as defined by the other dimension (e.g., orthographic neighbors).

In the current context of orthographic depth, we apply the same principle to quantify orthographic–phonological consistency, using a measure we henceforth label OPC. OPC is defined as the mean *phonological* similarity of a word to its *orthographic* neighbors. Given our interest in overall structure of the O-P mapping, not the consistency of individual words within a language (cf. Marelli & Amenta, 2018; Siegelman et al., 2022), we quantify mean OPC values across all monosyllabic words in each writing system (note that OPC can also be extended to polysyllabic words, however for comparability with other measures above we focus on monosyllabic words).

Practically, we define orthographic neighbors as words with an Orthographic Levenshtein distance of 1 from the orthographic form of a word. Then, we compute the *phonological* distance between each word and its *orthographic* neighbors, again using Levenshtein Distance, now using phonological forms (e.g., in a lexicon where the word *mint* has *mints, tint, mist, hint,* and *pint* as orthographic neighbors*,* it will have an OPC value of 1.2: This is because the first four neighbors have a phonological distance of 1 from *mint*, whereas *pint* has a phonological distance of 2). This provides an estimation of how similar a pronunciation of a word is to the pronunciation of its orthographic neighbors. Higher value means greater distance, which corresponds to less O-P systematicity, such that words that are similar orthographically are relatively dissimilar phonologically. All estimates below were conducted on a subset of 1000 words sampled per language.^2^ Mean OPC values for each writing system are provided in Table 6.

**Table 6:** Mean distance-based orthographic–phonological consistency (OPC)

Language	Mean OPC
Czech	1.06
Danish	1.39
Dutch	1.12
English	1.26
French	0.89
German	1.14
Norwegian	1.27
Swedish	1.23

The OPC estimates pass a sanity check: In line with the previous results, Czech emerges as a shallow orthography with a close-to-one-to-one relationship, while the Germanic orthographies, including English and Danish, show high values. A counter-intuitive finding is the French value, which is actually below the value of 1. This is likely to be a result of a large number of homophones: due to the presence of silent letters, words which differ in one letter only are likely to have an identical pronunciation, and thus a phonological Levenshtein distance of 0 (e.g., *chat* [cat]–*chats* [cats], both pronounced as /ʃa/). It is noteworthy that French is often considered to be shallow in the orthography-to-phonology direction, but not in the phonology-to-orthography direction, as there is a limited number of ways to pronounce a written word, but many ways to spell a spoken word (Ziegler et al., 1997).

### Examining the interrelations between all measures

In the previous sections, we have introduced a large number of measures. In this section we ask: How do these measures relate to each other? Do they jointly form any theoretically meaningful dimensions of orthographic depth? As noted above, orthographic depth is unlikely to reflect a single dimension (Schmalz et al., 2015); examining the interrelations between the measures may tell us something about the underlying structure of this umbrella term.

Table 7 shows the correlations between all measures. As the sample size is very small (8 languages), these results should be treated as descriptive only. Interestingly, different measures seem to cluster together, producing particularly high correlations with each other. In particular, measures of vowel consistency, body consistency, vowel entropy at the three grain sizes, the percent of irregular words according to the DRC rules, all show considerable correlations with each other (|*r*| > 0.7). Conversely, measures including the percent of words with identical length, number of GPCs, and number of multiletter rules seem to form their own cluster of variables (again, showing high correlations with each other, |*r*| > 0.6, while showing considerably lower correlations with the variables in the first group). This already supports the notion that different groups of measures tap into separable dimensions of orthographic depth, with the first set of measures indexing unpredictability and the second set indexing complexity.

**Table 7:** Correlations between different suggested measures of orthographic depth (*N* = 8 languages)

	1	2	3	4	5	6	7	8	9	10	11	12	13	14	15
1) *N* Unique GPCs		−0.79	−0.27	0.78	0.87	−0.03	−0.11	−0.25	0.15	0.11	0.18	−0.81	−0.31	0.01	0.61
2) % words: Same *N* of letters and phonemes			0.16	−0.86	−0.5	0.29	−0.11	0.04	0.11	0.15	0.06	0.87	0.14	0.4	−0.5
3) *N* single-letter rules				−0.09	−0.13	−0.58	0.93	0.76	−0.89	−0.9	−0.7	0.12	0.46	−0.62	−0.82
4) *N* multiletter rules					0.64	−0.19	0.13	−0.12	−0.11	−0.19	0.01	−0.58	−0.38	−0.46	0.26
5) *N* context-sensitive rules						−0.04	−0.09	−0.23	0.13	0.12	0.19	−0.52	−0.32	0.12	0.36
6) % irregular words							−0.79	−0.9	0.85	0.77	0.94	0.17	−0.8	0.76	0.44
7) Vowel consistency								0.87	−0.99	−0.99	−0.87	−0.04	0.58	−0.83	−0.73
8) Body consistency									−0.92	−0.85	−0.99	0.07	0.89	−0.7	−0.65
9) Entropy: Context-independent										0.98	0.92	0.01	−0.65	0.85	0.73
10) Entropy: Onset-conditional											0.85	0.03	−0.52	0.88	0.73
11) Entropy: Coda-conditional												−0.03	−0.87	0.76	0.61
12) Entropy accounted (letters)													0.06	0.11	−0.64
13) Entropy accounted (graphemes)														−0.38	−0.39
14) Mean OPC															0.54
15) Onset entropy															

**Fig. 1:**
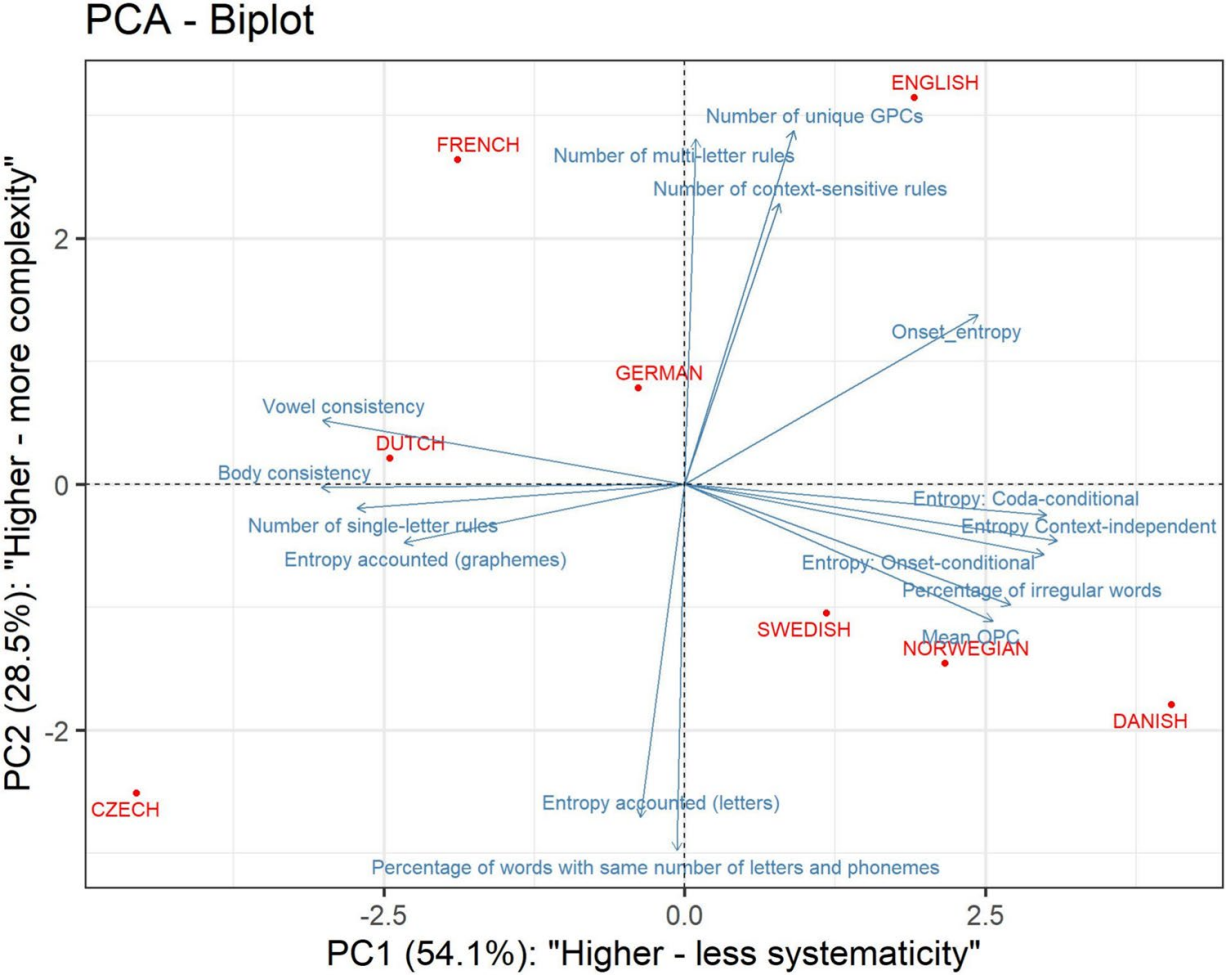
PCA biplot showing the loadings of the measures and position of each orthography

To further examine whether and how our measures group together, we next ran a Principal Component Analysis (PCA). The results of this analysis—which again, should be looked at only descriptively given the very limited sample size (*N* = 8 languages)—are shown in Fig. 1.

As can be seen in Fig. 1, the two components of the PCA correspond to the two sets of intercorrelated variables identified above on the basis of the correlation table. The variables that load on the first component (the horizontal axis in Fig. 1) are measures that reflect the systematicity of the mapping of orthographic units (whatever their size) to phonology, including the three entropy measures, the two consistency measures, the percent of irregular words per DRC, the number of single letter GPC rules, mean OPC, and the entropy-reduced efficiency measure computed based on graphemes.^3^ In contrast, the variables that load on the second component (the vertical axis in Fig. 1) are measures that are sensitive to the size of the orthographic units that map into phonemes, including the number of GPCs, Number of multiletter and context-sensitive rules, percent of words with identical number of phonemes and graphemes, and the entropy-reduced efficiency measure computed based on letters. Thus, the results of the PCA are consistent with Schmalz et al.’s (2015) proposal that orthograph depth involves two major dimensions: unpredictability, which is equivalent to what we now refer to as systematicity, and complexity, which can also be operationalized as the size of units. The only measure loading equally on both dimensions is Borgwaldt’s onset entropy. Theoretically, this measure picks up both on unpredictability (e.g., pairs such as “gift”–“gist” increase entropy, because of the different pronunciations of the first letters *g*) but also predictable complexities (e.g., “seat”–“sheet”—even though the different first phonemes of these two words are predictable, as the letter *s* is part of the multiletter grapheme *sh* in the latter case). Furthermore, onset entropy is not sensitive to irregularities that occur either at the level of vowels (as is common in English and Danish) or final consonants (as is common in French). Nevertheless, onset entropy correlates highly with the vowel entropy measures (r > 0.6) and may thus be a very useful rough indicator of an orthography’s classification in terms of the broader combined concept of orthographic depth.

Figure 1 also presents the distribution of the 8 languages in relation to the two first components of the PCA. The relative location of the different languages largely follows intuitions as well as previous theoretical analyses of the properties of these writing systems. For example, Czech, which has a writing system with small units which are also mapped systematically onto speech, is the only language located in the bottom left corner of Fig. 1, reflective of high predictability and low complexity. Danish, a language with a writing system with relatively small units but extensive unpredictability, is located in the lower right quadrant (low systematicity and low complexity part). English, typically considered a deep orthography (in both dimensions), is indeed located in the top right quadrant (low systematicity, high complexity). Some other languages differ mostly in their predictability (e.g., Dutch and German, both having relatively similar complexity, but differing in their level of predictability, with Dutch being more predictable, as expected; Landerl & Reitsma, 2005). To be clear, the exact position of languages in this graph should be taken with a grain of salt, given the very small sample size this analysis is based on. Still, the fact that multiple meaningful patterns do emerge already from this small sample size is encouraging and potentially theoretically insightful. We return to the implication of this finding in the General Discussion.

## General discussion

Above, we present four classes of measures of orthographic depth for eight alphabetic orthographies. To our knowledge, we provide the most comprehensive evaluation of orthographic depth to date, in terms of both the number of orthographies and the number of quantification methods. This is an important step towards understanding various orthography-level characteristics that affect reading processes and moving beyond anglocentricity in reading research (Huettig & Ferreira, 2022; Share, 2008).

Using a variety of approaches, varying in their theoretical assumptions, we have obtained results that are in line with the proposed division between complexity and unpredictability (Schmalz et al., 2015): that is, the extent to which one can benefit from relying on larger units, and the degree of systematicity in which these orthographic units map onto phonology^4^. We have furthermore extended previous analyses by including Scandinavian orthographies. This allows us to show a double dissociation: In Fig. 1, each quadrant contains at least one orthography. This suggests that, while complexity and unpredictability are correlated, we can find orthographies that are low on complexity and high on unpredictability (Danish) and low on unpredictability and high in complexity (French). This provides a justification for empirical studies which would aim to identify cognitive and behavioral consequences of each of the dimensions separately. Previous studies have either used artificial orthographies to dissociate the behavioral consequences of complexity versus unpredictability (Schmalz et al., 2022), or relied on French as the only orthography where complexity and unpredictability can be dissociated (De Simone et al., 2021; Schmalz et al., 2016).

Two of the four classes of measures we employed are new in the context of orthographic depth: efficiency and distance-based measures. Efficiency measures are obtained by calculating mutual information between orthographic and phonological forms. Efficiency measures rely on the same rationale as consistency and entropy measures but aim to overcome the reliance on certain assumptions that underlie these measures. For both consistency and entropy, one needs to decide on the unit which one wants to focus on (e.g., body-rime for body consistency, first letter and phoneme for onset entropy). Although we can, theoretically, calculate consistency or entropy for all units of the word (e.g., for each grapheme–phoneme-correspondence), we are faced with multiple ways to combine the consistency values within a word into a single number: for example, we could either sum or average the consistency for a given word. Mutual information and efficiency can be calculated for a whole word. Furthermore, when letters and phonemes are used as the basic units, we minimize the number of assumptions that go into the calculations: If we were to use the more classical measures of body consistency or vowel entropy, we need to make implicit assumptions about psychologically important units.

The distance-based measure, Orthography-Phonology-Consistency (OPC), is inspired by the Orthographic-Semantic-Consistency measure (Marelli & Amenta, 2018; Marelli et al., 2015), as we apply the same logic to quantifying the relationship between orthography and phonology: in a shallow orthography, we assume that words that are orthographically similar are also phonologically similar. Again, with this approach we minimize the assumptions behind the measure: as the basic units for calculating the measure are letters and phonemes, we do not make any additional assumptions about psychologically relevant sublexical measures. While efficiency still relies on the concept of consistency and thus is more closely linked to the connectionist theoretical framework, OPC does not rely on the concept of rules or statistics and is thus more theory-neutral (note that the “C” in “OPC” is taken by analogy of the OSC measure, and does not reflect a commitment to any theoretical framework).

However, being theoretically neutral comes both with advantages and disadvantages. On the one hand, measures that have been developed without a commitment to a particular theoretical framework are more likely to be generalizable. On the other hand, relying on models from cognitive science allows us to derive measures that are more likely to affect processes with psychological reality. Contrasting approaches that are based on different theories can, in principle, test differential empirical predictions and provide theoretical insights. In this particular case, however, there is high agreement between different approaches, thus making such a comparison unfeasible.

While it is possible to devise measures with a goal of minimizing the number of assumptions, all measures rely on some assumptions that may or may not hold up. For all measures that we introduced, an underlying assumption is the importance of letters and phonemes in visual word recognition; some measures additionally require the assumption of psychological reality of graphemes (Marinus & de Jong, 2011; Perry, 2023; Rey et al., 2000) or bodies (Schmalz et al., 2014). For the efficiency measures, we checked the robustness of the letter assumption by using letter bigrams and biphones as the relevant units. This is a computationally easy robustness check. Relying on graphemes as opposed to letters or bigrams requires assumptions about what constitutes a grapheme, and at least semimanual coding of each word’s division.

On the phonological level, some language-specific issues arise in defining phonemes. For example, some Czech consonants can function as a vocalic consonant (e.g., the letter *r*, which is a consonant in *krok* [/krɔk/, meaning “step”], and a vocalic consonant in *krk* [/kr̩k/, meaning “throat”]. According to IPA conventions, /r/ and /r̩/ as a vocalic consonant are separate phonemes; however, they do not meet other definitions’ criteria for being considered as separate phonemes (e.g., there are no minimal word pairs where the difference between /r/ and /r̩/ would lead to a difference in meaning). Thus, the theorist needs to make a decision whether to treat these two phonemes as being the same or different. Such differences in the definition of a phoneme can affect the estimate of depth, as this decision would determine whether a given orthographic unit can map onto only one phoneme, or two. Although our analyses focused on monosyllabic words, similar issues are relevant to analyses involving complex words, such as defining syllable boundaries or deciding which orthographic sequences correspond to morphemes.

A pragmatic question that falls out of the data we present is: which of our measures is the best? We cannot give a definite answer, partly because of the strong correlations among these measures, and partly because the ‘best’ quantification may depend on the researcher’s aim and other pragmatic considerations. The measures differ from each other in terms of:

1. Theoretical assumptions,
2. Computational convenience and automatability,
3. Extendibility to polysyllabic words,
4. Extendibility across languages, and
5. The possibility of calculating the measure not only on the language level, but also on the level of individual words or even orthographic units.

It is worth highlighting the high correlations that we found within the two clusters formed by the different measures. Again, given the small number of languages, these results should be confirmed by larger studies; however, as a preliminary finding, they are comforting, because they suggest that it might not make a huge difference which measure is chosen (as long as the distinction between complexity and systematicity is considered). We confirm prior theoretical work by showing that a single dimension called orthographic depth is unlikely to exist, and that we need to distinguish between complexity and unpredictability. However, within the clusters of complexity and unpredictability, we do not provide empirical evidence for the superiority of any given measure. As far as we can tell with the available data, researchers can opt to rely on the measures which are most easily calculated: For complexity, the easiest measure by far is the proportion of words with the same number of letters and phonemes: if a researcher has access to a corpus with orthographic and phonological word forms, they can even use a standard spreadsheet software to calculate the length of the entry in each cell, divide the length of the orthographic word form by the length of the phonological wordform, and count the proportion of entries where the resulting value equals to one. For unpredictability, the OPC measure is the easiest to calculate in the sense that it does not require any semimanual specification of sublexical units. That said, it is important to highlight that these conclusions are based on a small number of alphabetic European languages, and on monosyllabic words only. It is possible that when more diverse input is considered, important differences between the metrics of depth would emerge.

The current study provides the basis for future linguistic and behavioral work. As a first step, future research needs to overcome some of our methodological limitations. First, we were limited by available corpora. The quality of corpora is likely to vary across languages, with traditionally well-studied languages like English benefiting from higher-quality and more extensive corpora (Chilson et al., 2024). Guaranteeing the comparability of corpora is challenging, because the different datasets have also been collected for different purposes. CELEX and Lexique 2 were created by psycholinguists, for research purposes. The Czech corpus focusses on child language, whereas the NST lexicon aims to provide a reflection of actual language use. The differences in register between the corpora may systematically skew the results: for example, while the status of the Czech orthography as extremely shallow is in line with previous descriptions of the language (Caravolas, 2017), our analyses may exaggerate its position. Words used by children are shorter and less likely to be loanwords, which may lead to lower estimates of depth. Concerning the Scandinavian orthographies, conversely, the source of the corpora may systematically increase the estimates of depth. As the aim of those corpora was to capture spoken language, dialectal variations may bias the depth estimates due to untypical pronunciations.

In addition to the register, the size of the corpus may systematically affect the orthographic depth estimate: large corpora include more low-frequency words. These, in turn, are more likely to include jargon and loanwords (e.g., “precedent,” “anemone,” “bouquet”), which is more likely to be derived from Romance languages and thus follow different grapheme–phoneme-conversion rules than higher-frequency words (Hernandez et al., 2021). Furthermore, the register of the corpus matters: if the corpus contains colloquial speech, it may contain some dialectal words that, again, follow different grapheme–phoneme conventions than more formal, written texts. Here, we relied on existing resources, which have been published independently of one another. While some multilingual resources exist (e.g., Niekler et al., 2017), it is critical, for our purposes, to have phonological transcriptions of all items, which is a rarity in existing resources. Efforts are underway to create a multilingual corpus with IPA transcriptions (Chilson et al., 2024), but further validation of the corpus data is required before examining the robustness of our current analyses or extending the current work to further orthographies.

Second, we considered only monosyllabic words. The focus on monosyllabic words has been criticized about previous attempts to quantify depth, as there are differences across languages in the amount and percentage of words that are monosyllabic, with the English language being particularly rich in monosyllabic words (Protopapas & Vlahou, 2009). Many of our measures can only be applied to monosyllabic words, such as all DRC-derived measures and body-rime and vowel consistency and entropy. The latter two can be, in principle, extended to polysyllabic words. However, this would require an arbitrary decision about how to calculate a single consistency measure, for example, by averaging or summing the inconsistency across all vowels in the word. Other measures can be in principle extended to other orthographies. These include onset entropy (which has already been applied to polysyllabic words; Borgwaldt et al., 2004) and the novel measures, mutual information and OSC. There are methodological and theoretical challenges that prevent us from extending the current calculations to polysyllabic words at this stage. From a methodological perspective, the unevenness in corpora, as described above, would pose an exacerbated issue if we include polysyllabic words in our analysis. Polysyllabic words tend to be lower-frequency than monosyllabic words. Thus, depending on the size of the corpus, for example, the number of extremely low-frequency jargon and loan words may differ across languages. Such words, as mentioned above, may not follow the letter-sound rules of the orthography, thus systematically biassing the depth estimates. Longer words may also differ systematically between languages for a wide range of additional reasons: for example, Germanic languages and German in particular are known for their productivity in compounding words. Without a thorough examination of how such factors affect and interact with orthographic depth, values may be difficult to interpret. From a theoretical perspective, there is a relationship between the number of morphemes and the number of syllables. Although multimorphemic words can be monosyllabic (e.g., “walked”), including polysyllabic words would substantially increase the number of morphologically complex words, and thus numerous derivations of the same lemma. As words that are morphologically related are likely to also have similar spellings, this could systematically decrease the depth estimate for morphologically rich orthographies, as they would have a higher number of morphologically related word forms.

As a third methodological limitation, we have focused only on 8 orthographies. Providing depth estimates for a larger number of orthographies would enable both linguistic and behavioral comparisons involving a larger set of languages and orthographies. Furthermore, it would also address the important issue of the generalizability of these measures. Here, all are inflectional languages written in alphabetic scripts. As such, our work is but one step towards breaking the bias towards alphabetic languages that is present in reading research (Blasi et al., 2022; Huettig & Ferreira, 2022; Share, 2014). Some of the measures of orthographic depth we consider are not directly transferable to most nonalphabetic orthographies, where the mapping between different units may be qualitatively different. Furthermore, other dimensions might be more relevant to explaining cross-linguistic differences in reading. This warrants future theoretical and empirical investigation.

One potential consequence of considering a broader range of orthographies is that this could reveal limitations in the applicability of some of the measures. All our measures can be, in principle, applied to other alphabetic orthographies with relatively minor adjustments. However, they may not be equally meaningful. First, some of our measures, such as body consistency, are not trivially generalizable beyond monosyllabic words, and rely on a phonological structure that may have limited psychological reality in non-European languages (Kim, 2008; Share & Blum, 2005). Body consistency for monosyllabic words can be calculated in a semiautomated way, providing that the programmer specifies which letters in the alphabet are vowels and which are consonants. The proportion of words that are monosyllabic differs across languages (Protopapas & Vlahou, 2009); when monosyllabic words are rare, the measures will describe only a fraction of the orthography. Thus, an initial investigation should also examine to what extent the measures we presented here are extendable to polysyllabic words. Promising candidates are OPC and efficiency. Onset entropy has also been developed with the aim of providing a measure that is equally applicable to monosyllabic and polysyllabic words, but it has the drawback of focusing only on the initial letter and phoneme of a word (and, as noted above, estimates of onset entropy may not be well suited to differentiate between predictability and complexity).

Future research can investigate further features related to the orthography that affect reading and spelling processes. For example, some of our proposed measures can be used to measure the consistency both in the print-to-speech and in the speech-to-print direction. If there are multiple ways to spell a given phoneme, this may have behavioral consequences when it comes to writing skills (e.g., Galuschka et al., 2020). Furthermore, the closeness between print and speech sounds may vary due to factors other than the relationship between graphemes and phonemes, yet we focus on segmental features only. Unpredictability or quasi-regularity in print-to-speech translation may occur, for example, on the level of stress assignment (Jouravlev & Lupker, 2015; Sulpizio et al., 2013). This is not equally true for all languages: in French, lexical stress consistently falls on the last syllable. Across languages, the cues that determine lexical stress are also likely to differ. In some cases, stress is determined by simple rules, such as in French, or in the case of explicit markers for final-syllable-stress words in Italian (e.g., città, meaning city). At other times, more complex regularities determine lexical stress, such as similarity to other words in Italian and reduction in inconsistency within word classes in Russian (Jouravlev & Lupker, 2015; Sulpizio et al., 2013). Other non-segmental features may apply only to single orthographies. In the current analysis, we have removed the super-segmental feature of stød in Danish, which refers to a laryngealization in the production of certain words. An in-depth (no pun intended) qualitative analysis of each language may help us to identify other features that differ across languages and that may have behavioral consequences.

The issues above require further linguistic work. Some open questions about the behavioral level can already be posed based on our results. First, relatively little work examines the effects of different dimensions of orthographic depth on the cognitive level (but see De Simone et al., 2021; Schmalz et al., 2016, 2022). While previous research has identified French as a complex, yet predictable orthography, we have identified Danish as a simple, but unpredictable orthography. Thus, a study focusing on four orthographies falling in different quadrants of the complexity-unpredictability space would allow us to differentiate the effects of these two dimensions. Future research can use a similar PCA approach based on a large number of variables and a greater number of languages to further inspect how different orthographies are dispersed along the space of orthographic depth.

The measures provided in the current article can inform controlled experiments on cognitive processing underlying reading in adults. Such studies can, in turn, help us to refine the measures, by identifying whether behavioral outcomes are more closely related to one measure over another. This, again, will provide theoretical insights, as we can adjust the assumptions that go into the calculations of the measures, for example, about the orthographic units that have psychological reality.

To conclude, we argue that measuring orthographic depth requires a consideration of its operationalization. At this stage, there are multiple valid ways of operationalizing orthographic depth, and thus multiple meaningful ways of measuring it. To answer the question from this articles title: We propose that orthographic depth can and should be measured, in principle, and that a consideration of the operationalization of orthographic depth in a given context is critical to justifying the choice of measurement.

## Data Availability

Not applicable.

## Footnotes

1 An alternative approach is to map the remaining letters to a null phoneme. This approach tends to increase the overall level of entropy associated with the pronunciation of each level, but the qualitative pattern of mutual information across languages remains the same.

2 The subsampling was done due to considerations of computational efficiency and to mitigate potential issues related to different corpus sizes in the different languages. Further analyses revealed that a subsampling of 1,000 words is enough to result in stable estimates of mean OPC: resampling 1000 words randomly produces estimates within 0.02 units from the values reported below for all eight languages.

3 Due to differences in sign, some measures are negatively loaded on this component (e.g., consistency measures, where higher means more systematicity) and some are positively loaded on it (e.g., entropy measures, where higher means less systematicity). The same is true for the second component reflecting complexity.

4 We refer to “predictability” as the operationalisation of the concept introduced by Schmalz et al. (2015), and “systematicity” as the underlying concept, which is independent of a theoretical framework.

## Code availability

The code and supplementary materials are available at: https://osf.io/fmxy8/
